# Crystal structures of the silver iodide sulfates Ag_3_ISO_4_ and Ag_4_I_2_SO_4_

**DOI:** 10.1107/S2056989025008898

**Published:** 2025-10-31

**Authors:** Yuta Matsushima, Kento Uchida, Ryota Kawanago, Mizuki Yamamoto, Hisanori Yamane

**Affiliations:** a Applied Chemistry Chemical Engineering and Biochemical Engineering Yamagata, University, 4-3-16 Jonan Yonezawa-shi Yamagata 992-8510 Japan; b Institute of Multidisciplinary Research for Advanced Materials Tohoku, University, 2-1-1 Katahira Aoba-ku Sendai 980- 8577 Japan; Vienna University of Technology, Austria

**Keywords:** silver iodide sulfate, ionic conductivity, Ag^+^ Ag^+^ inter­action, crystal structure

## Abstract

The crystal structures of two phases in the AgI–Ag_2_SO_4_ system, Ag_3_ISO_4_(AgI:Ag_2_SO_4_ = 1:1; systematic name: tris­ilver iodide sulfate), and Ag_4_I_2_SO_4_ (AgI:Ag_2_SO_4_ = 2:1; systematic name: tetra­silver diiodide sulfate), were determined by single-crystal X-ray diffraction. The crystal structure model of Ag_3_ISO_4_ contains triangularly arranged Ag atoms in zigzag ladder chains extending parallel to the *a* axis. The Ag zigzag ladder chains alternate with rows of SO_4_ groups, while iodine atoms are accommodated within the concavities of the zigzag ladders. The crystal structure of Ag_4_I_2_SO_4_ was refined using a split-atom model for two of the four Ag sites. The disordered Ag sites are situated between the layers containing SO_4_ groups parallel to the *ac* plane, whereas the Ag sites within the SO_4_ layers are fully occupied and exhibit no disorder. The positional disorder of some of the Ag sites is considered to be associated with the moderate ionic conductivity of Ag_4_I_2_SO_4_, approximately 10^−3^ S cm^−1^ at room temperature. This is the first report describing the crystal structures of compounds containing Ag^+^, SO_4_^2–^ and I^−^.

## Abstract 2

The two compounds Ag_3_ISO_4_ and Ag_4_I_2_SO_4_, comprising Ag and I atoms and SO_4_ tetra­hedra, exhibit characteristic silver arrangements, with Ag_3_ISO_4_ containing triangularly arranged Ag atoms in zigzag ladder chains along the *a-*axis direction and Ag_4_I_2_SO_4_ featuring discrete Ag_4_ clusters.

## 1. Chemical context

α-Silver iodide (α-AgI) has been known as an Ag^+^ superionic conductor. The superionic conductive phase of AgI arises above the phase transition temperature of 420 K, and the ionic conductivity reaches a few S cm^−1^ (Tubandt & Lorenz, 1914 ▸; Boyce & Huberman, 1979 ▸). α-AgI undergoes a phase transition to the *β* phase when the temperature is decreased, and the superionic conductivity is lost on the phase transition. The superionic conductivity of Ag^+^ at room temperature (RT) has been reported in ternary phases of AgI and alkali halides such as Ag_4_RbI_5_ (Owens & Argue, 1970 ▸, 1967 ▸; Geller, 1967 ▸; Bradley & Greene, 1967*a* ▸,*b* ▸), Ag_4_KI_5_ (Owens & Argue, 1967 ▸; Bradley & Greene, 1966 ▸, 1967*a* ▸,*b* ▸), and Ag_3_KI_4_ (Takahashi *et al.*, 1970 ▸), and AgI and silver oxyacid salts in the glass states such as 0.8AgI–0.2(Ag_2_O–B_2_O_3_) (Chiodelli *et al.*, 1983 ▸), 0.58AgI–0.19Ag_2_O–0.23WO_3_ (Kuwano, 1990 ▸), 0.85AgI–0.15Ag_4_P_2_O_7_ (Minami *et al.*, 1977 ▸, 1980 ▸) and 0.75AgI–0.25Ag_2_MoO_4_ (Minami & Tanaka, 1980*a* ▸,*b* ▸), and in the crystalline state such as Ag_19_I_15_P_2_O_7_, Ag_7_I_4_PO_4_ (Takahashi *et al.*, 1972*a* ▸) and Ag_26_I_18_(WO_4_)_4_ (Chan & Geller, 1977 ▸). We have recently reported two crystalline compounds Ag_17_(CO_3_)_3_I_11_ (Watanabe *et al.*, 2021 ▸) and Ag_10_(CO_3_)_3_I_4_ (Suzuki *et al.*, 2021 ▸) in the AgI–Ag_2_CO_3_ system and found that the former, Ag_17_(CO_3_)_3_I_11_, is a superionic conductor with a conductivity of about 0.1 S/cm at RT.

According to the phase diagram of the AgI–Ag_2_SO_4_ system proposed by Takahashi *et al.* (1972*b* ▸), there is no stable crystalline phase with a specific composition. They reported an ionic conductivity of 5.0×10^−2^ S cm^−1^ in the glass phase with composition (1 − *x*) AgI–*x*(Ag_2_SO_4_) at *x* = 0.18–0.25. We synthesized new crystalline compounds at *x* = 0.5 (Ag_3_ISO_4_) and 0.33 (Ag_4_I_2_SO_4_). Polycrystalline bulk samples of Ag_3_ISO_4_ and Ag_4_I_2_SO_4_ prepared at 443 and 417 K had ionic conductivities of 9.5×10^−6^ and 9.2×10^−4^ S cm^−1^ at RT, respectively. These values are higher or comparable with those of Ag_13_(AsO_4_)_3_I_4_ (6.4×10^−6^ S cm^−1^ at 303 K; Pitzschke *et al.*, 2009*a* ▸) and Ag_10_(CO_3_)_3_I_4_ (4.4×10^−6^ S cm^−1^ at RT; Suzuki *et al.*, 2021 ▸). The ionic conductivities of Ag_3_ISO_4_ and Ag_4_I_2_SO_4_ are not necessarily high compared to the Ag^+^ superionic conductors, but these compounds are important as precursors of superionic conductors in the (1 − *x*) AgI–*x*(Ag_2_SO_4_) system.

## 2. Structural commentary

**Figure 1:**
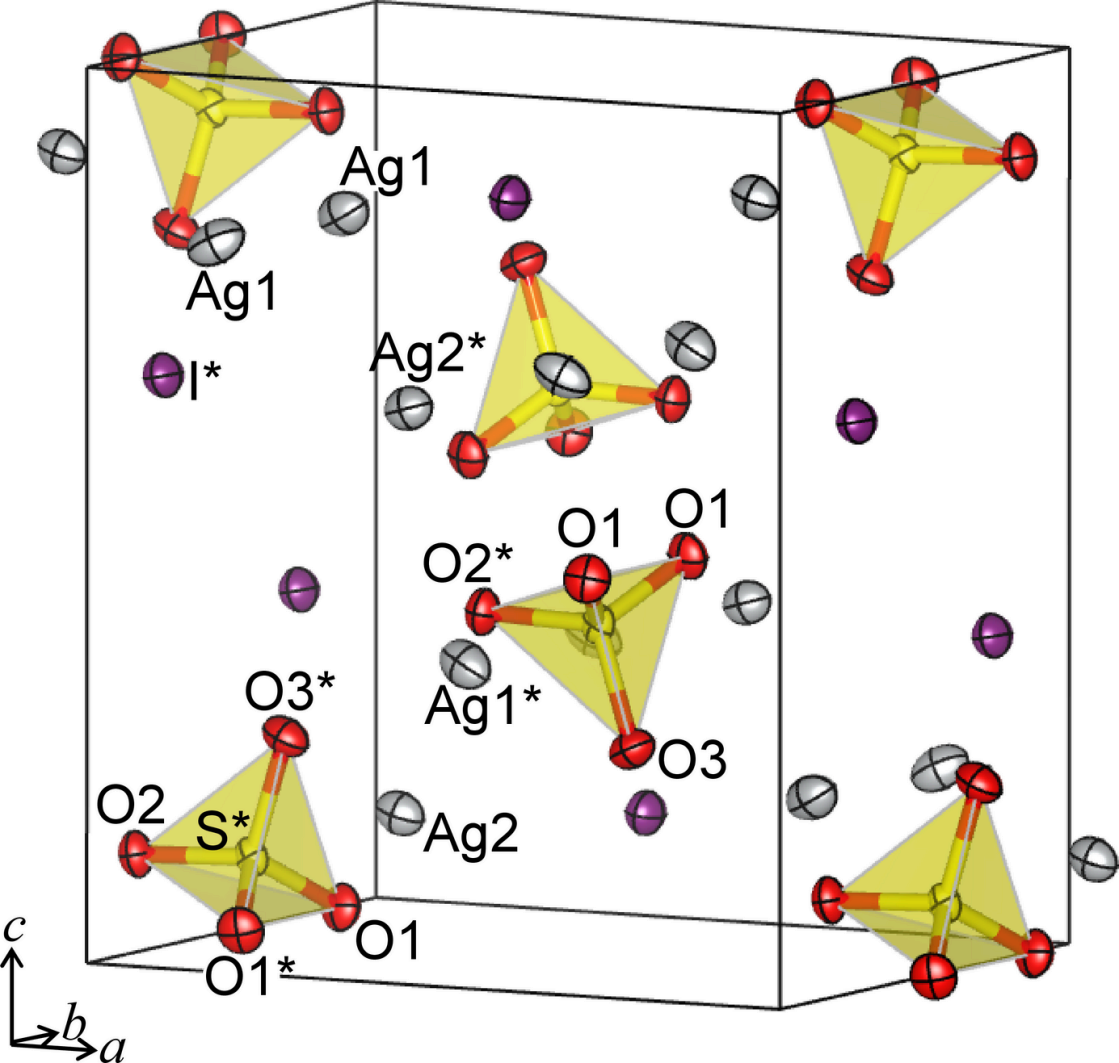
Perspective view of the crystal structure of Ag_3_ISO_4_. The probability of the anisotropic displacement ellipsoids is 50%. The asterisk denotes atoms located within the asymmetric unit.

Ag_3_ISO_4_ and Ag_4_I_2_SO_4_ crystallize with ortho­rhom­bic symmetry in the space groups *Pnma* and *Pna*2_1_, respectively. Figs. 1 ▸ and 2 ▸ show perspective views of the crystal structures of Ag_3_ISO_4_ and Ag_4_I_2_SO_4_, and selected inter­atomic distances are collated in Tables 1 ▸ and 2 ▸.

Ag_3_ISO_4_ contains two Ag sites, one I site, one S site, and three O sites in the asymmetric unit; Ag_4_I_2_SO_4_ contains four Ag sites, two I sites, one S site, and four O sites. Among the four Ag sites in Ag_4_I_2_SO_4_, Ag1 and Ag3 are split into two and three sites, respectively, with occupancies of 0.74 (1) and 0.26 (1) for Ag1*A* and Ag1*B*, and 0.803 (4), 0.098 (4), and 0.099 (4) for Ag3*A*, Ag3*B*, and Ag3*C*. S atoms in both compounds form tetra­hedral SO_4_ groups, and their S—O distances are 1.474 (3) Å for two O1, 1.488 (4) Å for O2, and 1.475 (4) Å for O3 in Ag_3_ISO_4_ and 1.466 (6) Å for O1, 1.468 (5) Å for O2, 1.505 (5) Å for O3, and 1.469 (7) Å for O4 in Ag_4_I_2_SO_4_. These values are comparable to the bond lengths between S and O in the SO_4_ groups found in inorganic crystals, including minerals and compounds such as barite, BaSO_4_ (1.464–1.483 Å; Sawada & Takeuchi, 1990 ▸), Ag_2_SO_4_ (1.473 Å; Mehrotra *et al.*, 1978 ▸), Na_2_SO_4_ (1.479 Å; Hawthorne & Ferguson, 1975 ▸), and gypsum, CaSO_4_·2H_2_O (1.457–1.461 Å; Cole & Lancucki, 1974 ▸).

In Ag_3_ISO_4_, Ag1 and O1 occupy the Wykoff position 8*d* with site symmetry 1. Ag2, S, O2, O3, and I reside at the 4*c* position with site symmetry *m* perpendicular to the *b* axis. In Ag_4_I_2_SO_4_, all the atoms lie on a general position 4*a*.

The silver atoms in Ag_3_ISO_4_ and Ag_4_I_2_SO_4_ are surrounded by oxygen and iodine atoms, and the coordination environments are different between the two structures. All silver atoms in Ag_3_ISO_4_ are coordinated by three O atoms and one I atom. On the other hand, in Ag_4_I_2_SO_4_, Ag1 (split into Ag1*A* and Ag1*B*) is surrounded by three O atoms belonging to two SO_4_ groups and nearly coplanar by four I atoms; Ag2 is surrounded by three O atoms of three different SO_4_ groups and three I atoms; Ag3 (split into Ag3*A*, Ag3*B*, and Ag3*C*) is coordinated by two O atoms and three I atoms; Ag4 is coordinated in form of a distorted octa­hedron with four I atoms at the equatorial and two O atoms at the axial positions. Table 3 ▸ compares the shortest and average distances of Ag—O and Ag—I in Ag_3_ISO_4_ and Ag_4_I_2_SO_4_ with several silver compounds. Here, the averages were calculated for Ag—O distances shorter than ∼2.8 Å and for Ag—I shorter than ∼3.7 Å. These boundaries are based on the ionic radii of Ag^+^, O^2–^, and I^−^ (Shannon, 1976 ▸). In averaging, a weight was taken into account, and the weight corresponds to the number of bonds present in the unit cell, which is calculated by multiplying the site multiplicity and the occupancy. The shortest Ag—O distances range from 1.886 Å in Ag_16_I_12_P_2_O_7_ to 2.405 Å in Ag_2_SO_4_. The shortest Ag—I distances in Ag_3_ISO_4_ and Ag_4_I_2_SO_4_ are between the shortest Ag—I distance of 2.312 Å in Ag_26_I_18_(WO_4_)_4_ and 2.841 Å in Ag_3_I(NO_3_)_2_. The average values of Ag—O and Ag—I distances in the title compounds are comparable to those observed for the other silver compounds listed in Table 3 ▸; the average Ag—I distance in Ag_3_ISO_4_, 2.791 Å, is slightly shorter than those in the other compounds, such as 2.814 Å in *γ*-AgI and 2.835 Å in Ag_16_I_12_P_2_O_7_.

**Figure 3:**
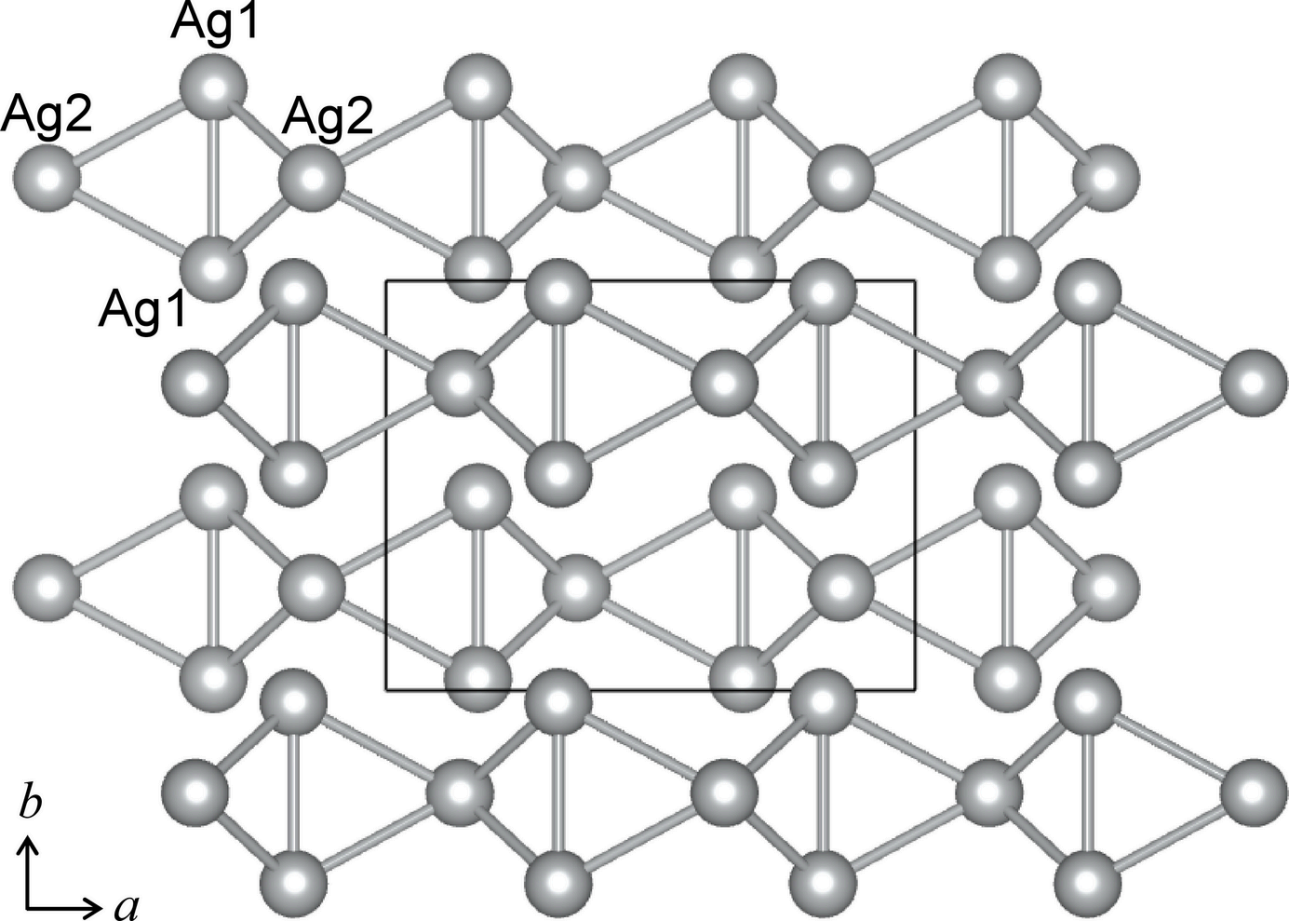
Arrangement of Ag atoms in Ag_3_ISO_4_. The linkers connect the Ag atoms within 3.3 Å for ease of visibility.

**Figure 4:**
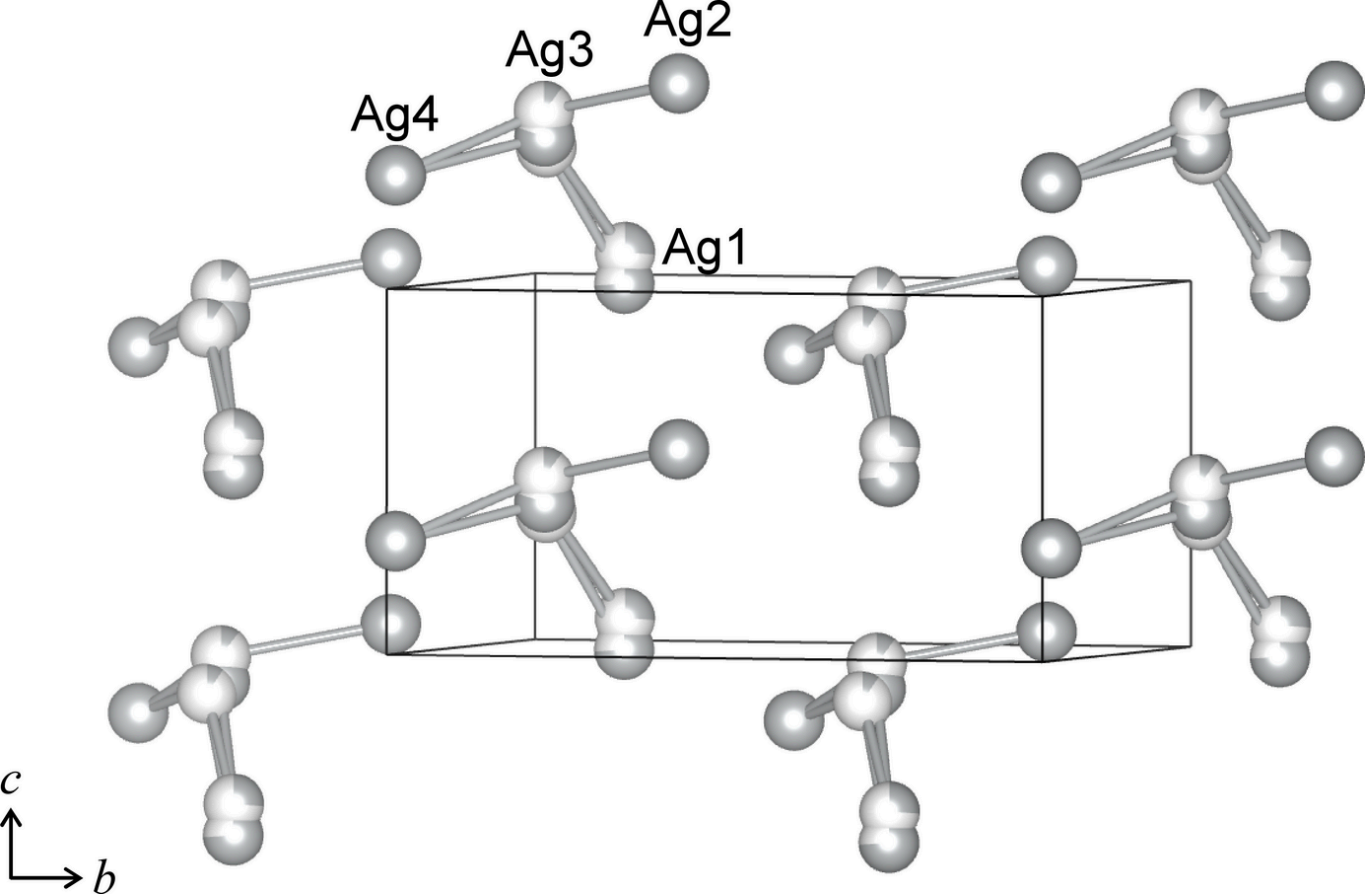
Arrangement of Ag atoms in Ag_4_I_2_SO_4_ (sliced view in the range *x* = 0.5–1.0). The linkers connect the Ag atoms within 3.3 Å for ease of visibility.

The shortest Ag—Ag distance of 2.9973 (6) Å is observed in Ag_3_ISO_4_ between Ag1 and Ag2 generated by the symmetry operation −*x* + 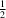, −*y* + 1, *z* − 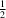. The second shortest Ag—Ag distance is 3.0604 (8) Å between the adjacent Ag1 sites. Ag_4_I_2_SO_4_ shows the shortest Ag—Ag distance between Ag1*B* and Ag3*B* of 2.805 (19) Å. These values are comparable to 2.873 Å in Ag_2_CO_3_ (Norby *et al.*, 2002 ▸), 2.880 Å in Ag_8_(CrO_4_)_3_I_2_ (Pitzschke *et al.*, 2009*b* ▸), and 2.942 Å in Ag_3_I(NO_3_)_2_ (Birnstock & Britton, 1970 ▸). These rather short Ag—Ag distances are considered to be due to the argentophilic inter­action between Ag^+^ ions in the *d*^10^ configuration, which often leads to characteristic arrangements of Ag in several inorganic crystals (Schmidbaur & Schier, 2015 ▸; Jansen, 1987 ▸). The arrangements of the silver atoms in the crystal structure of Ag_3_ISO_4_ and Ag_4_I_2_SO_4_, excluding the other atoms, are illustrated in Figs. 3 ▸ and 4 ▸, respectively. In Ag_3_ISO_4_, the silver atoms form triangles, which are connected to each other by sharing the Ag1—Ag1 edges and the Ag2 corners, comprising zigzag ladder chains extending parallel to the *a* axis (Fig. 3 ▸). Fig. S1 (electronic supplementary information) shows the projected views of the crystal structure of Ag_3_ISO_4_ along the *a* axis (*a*) and *b* axis (*b*). It shows that the rows of SO_4_ and the zigzag ladders of Ag are arranged alternately (*a*), and the iodine atoms are held in the concavities of the zigzag ladder chains (*b*). In Ag_4_I_2_SO_4_, the Ag atoms form Ag_4_ clusters, the arrangement of which in the crystal structure is shown in Fig. 4 ▸. The shortest and the second shortest I⋯I distances in Ag_4_I_2_SO_4_ are 4.2135 (10) nd 4.3301 (8) Å. By connecting I1 and I2, helical chains of iodine atoms along the *c* axis are recognized in Ag_4_I_2_SO_4_(Fig. S2). The Ag_4_ clusters and the SO_4_ groups in Ag_4_I_2_SO_4_ fill the space between the helical chains of I atoms.

## 3. Database survey

The Inorganic Crystal Structure Database (ICSD; Zagorac *et al.*, 2019 ▸) contains the crystal structure data for quaternary solid-state inorganic compounds comprising silver and iodide ions and oxyacid groups, such as Ag_3_I(NO_3_)_2_ (Birnstock & Britton, 1970 ▸), Ag_16_I_12_P_2_O_7_ (Garrett *et al.*, 1982 ▸), Ag_5_IP_2_O_7_ (Adams & Preusser, 1999 ▸), Ag_4_IPO_4_ (Oleneva *et al.*, 2008 ▸), Ag_8_(CrO_4_)_3_I_2_ (Pitzschke *et al.*, 2009*b* ▸), Ag_9_(GeO_4_)_2_I (Pitzschke *et al.*, 2009*c* ▸), Ag_8_I_4_V_2_O_7_ (Adams, 1996 ▸), Ag_13_(AsO_4_)_3_I_4_ (Pitzschke *et al.*, 2009*a* ▸), Ag_4_I_2_SeO_4_(Pitzschke *et al.*, 2008*a* ▸), Ag_3_ITeO_4_ (Pitzschke *et al.*, 2008*a* ▸), Ag_9_I_3_(IO_3_)_2_(SeO_4_)_2_ (Pitzschke *et al.*, 2008*b* ▸) and Ag_26_I_18_(WO_4_)_4_ (Chan & Geller, 1977 ▸). We have recently reported the crystal structures of two silver carbonate iodides with compositions of Ag_17_(CO_3_)_3_I_11_ (Watanabe *et al.*, 2021 ▸) and Ag_10_(CO_3_)_3_I_4_ (Suzuki *et al.*, 2021 ▸).

There are no data in the ICSD of a phase containing Ag^+^, I^−^, and SO_4_^2–^. Compounds containing I^−^ and SO_4_^2–^ in the crystal structure included in ICSD are (Pt(NH_3_)_4_)_2_I_2_(HSO_4_)_3_OH·H_2_O (Clark *et al.*, 1982 ▸), (Pt(NH_3_)_4_)(PtI_2_(NH_3_)_4_)(HSO_4_)_4_·2H_2_O (Tanaka *et al.*, 1986 ▸) and H(I(SO_4_)_2_) (Jansen & Müller, 1998 ▸).

## 4. Synthesis and crystallization

The starting material AgI was precipitated at 323 K in aqueous solutions of AgNO_3_ (99.8%, Kanto Chemical, Japan) and KI (99.5%, Kanto Chemical, Japan); Ag_2_SO_4_ was precipitated at RT in an aqueous solution of AgNO_3_ and 5-times diluted sulfuric acid (Kanto Chemical, Japan). The resulting AgI and Ag_2_SO_4_ powders were thoroughly mixed in an agate mortar at a molar ratio of 1:1 using a small amount of water as a mixing medium. The mixed powder was placed in a glass tube with one end open, heated in air at 433 K for 1 h, and cooled slowly to 408 K at a rate of 0.5 K/h. These conditions were determined after thermogravimetric-differential thermal analysis (TG-DTA) under constant flow of synthetic dry air (Fig. S3). Fig. S3 shows the TG-DTA curves of a mixture of AgI and Ag_2_SO_4_ powders in a 1:1 ratio. A sharp endothermic effect was observed at 431 K, corresponding to the melting point of this composition. The TG curve showed no substantial mass loss, indicating thermal stability in air up to 520 K. Upon cooling to RT, translucent pale brown lumps were obtained. Like other silver compounds, the silver iodide sulfates are moderately photosensitive, so the samples were treated in the dark under red light through the color filter from an LED lamp. Each fragment of the two types of crystals found in the lumps was fixed on glass fibers with an ep­oxy resin and mounted on a goniometer. The XRD data were collected at RT in the dark.

Powder samples of Ag_3_ISO_4_ and Ag_4_I_2_SO_4_ were prepared from AgI and Ag_2_SO_4_ to verify the validity of the crystal structure models determined by single crystal XRD, and the powder data were analyzed using the Rietveld method. Ag_2_SO_4_ was prepared from an aqueous solution of AgNO_3_ and sulfuric acid for the synthesis of Ag_3_ISO_4_, and from aqueous solutions of AgNO_3_ and Na_2_SO_4_ for the synthesis of Ag_4_I_2_SO_4_. AgI and Ag_2_SO_4_ were then mixed in molar ratios of 1:1 and 1:2, and heated in air at 414 K for 1 h to obtain Ag_3_ISO_4_, and at 411 K for 3 h to obtain Ag_4_I_2_SO_4_. For Ag_4_I_2_SO_4_, in order to reduce unreacted AgI and the by-product Ag_3_ISO_4_ phase, the heated mixture was slowly cooled to 373 K in the heater, followed by the repeated heat treatment after thorough grinding.

## 5. Refinement

Crystal data, data collection and structure refinement details are summarized in Table 4 ▸.

In the refined model of Ag_3_ISO_4_, the highest difference electron density peak of 1.18 e Å^−3^ is at a position 0.8646 (5) Å distant from I1. Two difference peaks of 1.10 and 1.03 e Å^−3^ remained at positions 0.7562 (5) and 0.7659 (4) Å distant from Ag1, and two peaks of 0.94 and 0.90 e Å^−3^ at positions 0.7782 (6) and 0.9591 (6) Å distant from Ag2. Difference peaks lower than 0.8 e Å^−3^ were detected near I1 (0.76 e Å^−3^), O3 (0.60 e Å^−3^), and Ag2 (0.55 e Å^−3^).

The crystal structure of Ag_4_I_2_SO_4_ was refined using a split-atom model for the Ag1 and Ag3 sites. For Ag1, 74.2% of the silver atoms occupy the Ag1*A* site, while the remaining 25.8% are distributed over the Ag1*B* site, located 0.595 (13) Å distant from Ag1*A*. For the Ag3 site, 80.3% of the silver atoms reside on the Ag3*A* site, with approximately 10% each distributed on the Ag3*B* and Ag3*C* sites, located 0.508 (17) and 0.49 (3) Å distant from Ag3*A*, respectively. Figure S4 presents a projection of the crystal structure of Ag_4_I_2_SO_4_ along the *a* axis. The disordered Ag1 and Ag3 sites are located between SO_4_ layers parallel to the *ac* plane, whereas the Ag2 and Ag4 sites within the SO_4_ layers are fully occupied. As described in section 1, Ag_4_I_2_SO_4_ exhibits a moderate ionic conductivity of 9.2×10^−4^ S cm^−1^ at RT, and the positional disorder of the Ag sites is considered to be associated with this conductivity. Furthermore, the disorder observed at the Ag1 and Ag3 sites suggests site-dependent contributions to Ag^+^ conduction. Specifically, Ag^+^ diffusion occurs preferentially within the inter­layer spaces between the SO_4_ layers via the Ag1 and Ag3 sites, whereas silver atoms at the Ag2 and Ag4 sites provide a minor contribution to Ag^+^ conduction. In the refined model of Ag_4_I_2_SO_4_, the highest difference electron density peak of 0.93 e Å^−3^ is near the Ag3 site, with distances between 0.800 (3) Å from Ag3*A* and 1.16 (2) Å from Ag3*C*. The second and fourth highest difference peaks (0.79 and 0.57 e Å^−3^) are at positions 0.8720 (7) and 0.7644 (7) Å distant from I1. The third-highest difference peak of 0.64 e Å^−3^ is found at (0.2880, 0.2611, 0.7269), corresponding to an inter­stitial position surrounded by two Ag atoms (Ag2 and Ag3), three I atoms, and O1. The distances are 1.86 (3) Å from Ag3*C*, 2.7660 (9) Å from Ag2, 2.6543 (7) and 2.6599 (7) Å from I1, 2.7182 (7) Å from I2, and 2.235 (7) Å from O1.

The structure models refined on basis of single crystal XRD data were verified by powder X-ray data using a Rietveld analysis (PDXL; Rigaku, 2018 ▸). The powder XRD data were collected using a Rigaku MiniFlex 600 powder X-ray diffractometer with Cu *K*α radiation (λ = 1.54183 Å) equipped with a 1D detector (Rigaku D/teX Ultra 250). The refinements were carried out with the atomic coordinates (*x*, *y*, *z*) and the atomic displacement parameters (*B*) fixed to those determined by the single crystal XRD study, and converged with *R*_wp_/*R*_p_/*S* = 5.31%/3.53%/3.98 for Ag_3_ISO_4_ and 3.06%/2.40%/1.33 for Ag_4_I_2_SO_4_ (Figure S5). The lattice parameters refined by the Rietveld analysis are *a* = 8.9437 (2), *b* = 6.9241 (2), and *c* = 10.2516 (2) Å for Ag_3_ISO_4_, and *a* = 9.2026 (4), *b* = 13.1072 (5), and *c* = 6.9545 (3) Å for Ag_4_I_2_SO_4_. These values are in good agreement with those obtained by the single crystal XRD analysis (Table 4 ▸).

## Supplementary Material

Crystal structure: contains datablock(s) global, Ag3ISO4, Ag4I2SO4. DOI: 10.1107/S2056989025008898/wm5769sup1.cif

Structure factors: contains datablock(s) Ag3ISO4. DOI: 10.1107/S2056989025008898/wm5769Ag3ISO4sup2.hkl

Rietveld powder data: contains datablock(s) Ag3ISO4. DOI: 10.1107/S2056989025008898/wm5769Ag3ISO4sup4.rtv

Structure factors: contains datablock(s) Ag4I2SO4. DOI: 10.1107/S2056989025008898/wm5769Ag4I2SO4sup3.hkl

Rietveld powder data: contains datablock(s) Ag4I2SO4. DOI: 10.1107/S2056989025008898/wm5769Ag4I2SO4sup5.rtv

CCDC references: 2494919, 2494918

Additional supporting information:  crystallographic information; 3D view; checkCIF report

## Figures and Tables

**Figure 2:**
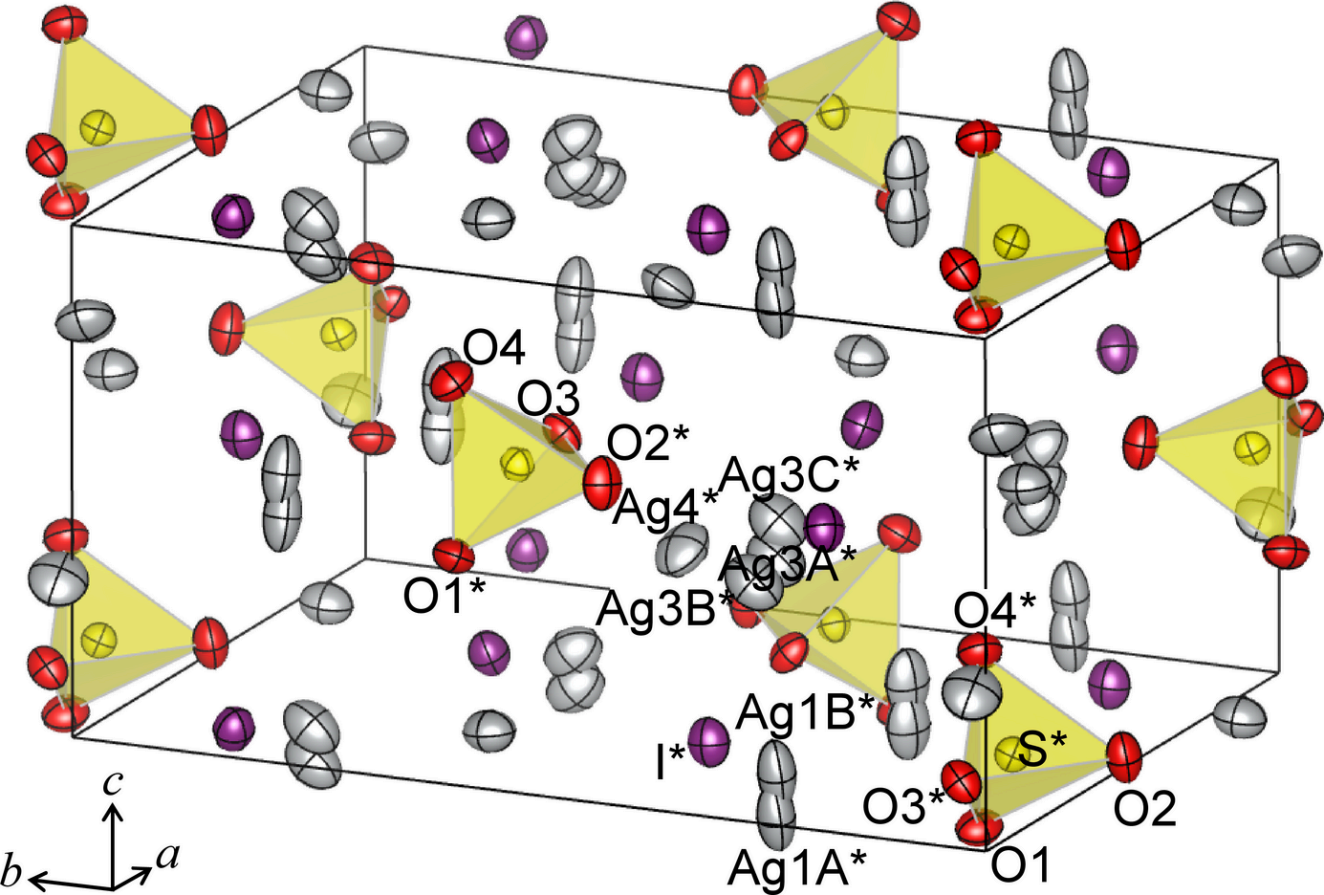
Perspective view of the crystal structure of Ag_4_I_2_SO_4_. The probability of the anisotropic displacement ellipsoids is 50%.. The asterisk denotes atoms located within the asymmetric unit.

**Table 1:** Selected bond lengths (Å) for Ag_3_ISO_4_

Ag1—O1^i^	2.384 (3)	Ag2—O1^i^	2.436 (3)
Ag1—O3	2.413 (3)	Ag2—O2	2.467 (4)
Ag1—O2	2.473 (3)	Ag2—I1^vi^	2.8066 (7)
Ag1—I1^ii^	2.7838 (5)	Ag2—I1	3.1714 (7)
Ag1—Ag2^ii^	2.9973 (6)	S1—O1^vii^	1.474 (3)
Ag1—Ag1^iii^	3.0604 (8)	S1—O1	1.474 (3)
Ag1—Ag2^iv^	3.2744 (6)	S1—O3	1.475 (4)
Ag2—O1^v^	2.436 (3)	S1—O2^viii^	1.488 (4)

Symmetry codes: (i) 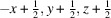; (ii) 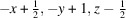; (iii) 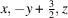; (iv) 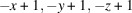; (v) 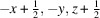; (vi) 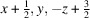; (vii) 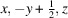; (viii) 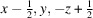.

**Table 2:** Selected bond lengths (Å) for Ag_4_I_2_SO_4_

Ag1*A*—O3	2.462 (5)	Ag3*A*—I1^v^	2.838 (3)
Ag1*A*—O1^i^	2.468 (7)	Ag3*A*—Ag4	3.184 (2)
Ag1*A*—I2	2.863 (2)	Ag3*B*—O2	2.375 (18)
Ag1*A*—I2^ii^	3.038 (2)	Ag3*B*—I2	2.608 (17)
Ag1*A*—I1^iii^	3.039 (5)	Ag3*B*—I1	2.686 (15)
Ag1*A*—Ag3*B*	3.256 (18)	Ag3*C*—O2	2.385 (19)
Ag1*B*—O3	2.412 (8)	Ag3*C*—I1^v^	2.620 (17)
Ag1*B*—O4^ii^	2.541 (10)	Ag3*C*—I1	2.694 (16)
Ag1*B*—Ag3*B*	2.805 (19)	Ag3*C*—I2	3.263 (17)
Ag1*B*—I2	2.889 (7)	Ag3*C*—Ag4	3.290 (16)
Ag1*B*—I2^ii^	3.039 (7)	Ag4—O3^v^	2.421 (6)
Ag1*B*—Ag3*A*	3.194 (10)	Ag4—O3^vi^	2.469 (7)
Ag2—O1	2.347 (6)	Ag4—I1^v^	3.0237 (13)
Ag2—O4^iv^	2.386 (7)	Ag4—I2	3.0879 (13)
Ag2—O2^i^	2.423 (5)	Ag4—I1^iv^	3.1247 (12)
Ag2—I2	2.8799 (9)	Ag4—I2^vii^	3.1741 (13)
Ag2—Ag3*C*^iv^	3.283 (18)	S1—O1^viii^	1.466 (6)
Ag3*A*—O2	2.522 (6)	S1—O2^viii^	1.468 (5)
Ag3*A*—I2	2.798 (3)	S1—O4	1.469 (7)
Ag3*A*—I1	2.8113 (19)	S1—O3	1.505 (5)

Symmetry codes: (i) 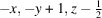; (ii) 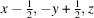; (iii) 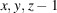; (iv) 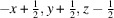; (v) 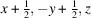; (vi) 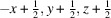; (vii) 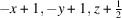; (viii) 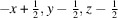.

**Table 3:** Comparison of inter­atomic distances (Å) between Ag atoms and anions in several silver compounds

Compound	Shortest Ag—O	Average Ag—O	Shortest Ag—I	Average Ag—I	Reference
Ag_3_ISO_4_	2.384	2.431	2.784	2.791	This work
Ag_4_I_2_SO_4_	2.346	2.564	2.607	3.034	This work
Ag_2_SO_4_	2.405	2.511	–	–	Mehrotra *et al.* (1978 ▸)
Ag_2_CO_3_	2.245	2.421	–	–	Norby *et al.* (2002 ▸)
Ag_10_(CO_3_)_3_I_4_^i^	2.252	2.437	2.714	3.004	Suzuki *et al.* (2021 ▸)
γ-AgI	–	–	2.814	2.814	Hull & Keen (1999 ▸)
Ag_13_(AsO_4_)_3_I_4_	2.293	2.375	2.708	3.071	Pitzschke *et al.* (2009*a* ▸)
Ag_26_I_18_(WO_4_)_4_	2.195	2.478	2.312	2.916	Chan & Geller (1977 ▸)
Ag_4_IPO_4_^ii^	2.278	2.368	2.706	3.034	Oleneva *et al.* (2008 ▸)
Ag_16_I_12_P_2_O_7_	1.886	2.340	2.680	2.835	Garrett *et al.* (1982 ▸)
Ag_5_IP_2_O_7_	2.274	2.466	2.765	2.948	Adams & Preusser (1999 ▸)
Ag_3_I(NO_3_)_2_	2.250	2.594	2.841	2.942	Birnstock & Britton (1970 ▸)
Ag_8_(CrO_4_)_3_I_2_^iii^	2.384	2.446	2.809	3.053	Pitzschke *et al.* (2009*b* ▸)
Ag_9_I_3_(IO_3_)_2_(SeO_4_)_2_	2.306	2.508	2.719	2.886	Pitzschke *et al.* (2008*b* ▸)
Ag_3_ITeO_4_	2.238	2.455	2.775	2.928	Pitzschke *et al.* (2008*a* ▸)
Ag_4_I_2_SeO_4_	2.322	2.508	2.822	3.069	Pitzschke *et al.* (2008*a* ▸)
Ag_8_I_4_V_2_O_7_	2.182	2.364	1.927	3.128	Adams (1996 ▸)
Ag_9_(GeO_4_)_2_I	2.106	2.222	3.364	3.431	Pitzschke *et al.* (2009*c* ▸)

Measurement temperatures: (i) 90 K, (ii) 173 K, (iii) 273 K.

**Table 4:** Experimental details

	Ag_3_ISO_4_	Ag_4_I_2_SO_4_
Crystal data		
Chemical formula	Ag_3_ISO_4_	Ag_4_I_2_SO_4_
*M*_r_	546.57	781.34
Crystal system, space group	Orthorhombic, *Pnma*	Orthorhombic, *Pna*2_1_
Temperature (K)	300	301
*a*, *b*, *c* (Å)	8.9418 (6), 6.9182 (5), 10.2660 (7)	9.2072 (3), 13.1007 (4), 6.9528 (2)
*V* (Å^3^)	635.07 (8)	838.65 (4)
*Z*	4	4
Radiation type	Mo *K*α	Mo *K*α
μ (mm^−1^)	14.28	16.77
Crystal size (mm)	0.05 × 0.04 × 0.04	0.10 × 0.03 × 0.01

Data collection		
Diffractometer	Bruker APEXII CCD	Bruker APEXII CCD
Absorption correction	Multi-scan (*SADABS*; Krause *et al.*, 2015 ▸)	Multi-scan (*SADABS*; Krause *et al.*, 2015 ▸)
*T*_min_, *T*_max_	0.615, 0.746	0.53, 0.75
No. of measured, independent and observed [*I* > 2σ(*I*)] reflections	16851, 790, 770	6220, 1806, 1768
*R*_int_	0.039	0.035
(sin θ/λ)_max_ (Å^−1^)	0.650	0.649

Refinement		
*R*[*F*^2^ > 2σ(*F*^2^)], *wR*(*F*^2^), *S*	0.020, 0.047, 1.15	0.022, 0.051, 1.08
No. of reflections	790	1806
No. of parameters	49	115
No. of restraints	0	2
Δρ_max_, Δρ_min_ (e Å^−3^)	1.18, −0.87	0.93, −0.79
Absolute structure	–	Refined as an inversion twin
Absolute structure parameter	–	0.35 (4)

Computer programs: *APEX3* and *SAINT* (Bruker, 2017 ▸), *SHELXT* (Sheldrick, 2015*a* ▸), *SHELXL* (Sheldrick, 2015*b* ▸), *VESTA* (Momma & Izumi, 2011 ▸) and *publCIF* (Westrip, 2010 ▸).

## supplementary crystallographic information

### Trisilver iodide sulfate (Ag3ISO4) Crystal data

**Table:**

Ag_3_ISO_4_	*D*_x_ = 5.717 Mg m^−^^3^
*M**_r_* = 546.57	Mo *K*α radiation, λ = 0.71073 Å
Orthorhombic, *P**n**m**a*	Cell parameters from 9985 reflections
*a* = 8.9418 (6) Å	θ = 3.0–27.5°
*b* = 6.9182 (5) Å	µ = 14.28 mm^−^^1^
*c* = 10.2660 (7) Å	*T* = 300 K
*V* = 635.07 (8) Å^3^	Block, translucent colourless-brown
*Z* = 4	0.05 × 0.04 × 0.04 mm
*F*(000) = 968	

### Trisilver iodide sulfate (Ag3ISO4) Data collection

**Table:**

Bruker APEXII CCD diffractometer	790 independent reflections
Radiation source: micro focus sealed tube	770 reflections with *I* > 2σ(*I*)
Detector resolution: 7.3910 pixels mm^-1^	*R*_int_ = 0.039
φ and ω scans	θ_max_ = 27.5°, θ_min_ = 3.0°
Absorption correction: multi-scan (*SADABS*; Krause *et al.*, 2015)	*h* = −11→11
*T*_min_ = 0.615, *T*_max_ = 0.746	*k* = −8→8
16851 measured reflections	*l* = −13→13

### Trisilver iodide sulfate (Ag3ISO4) Refinement

**Table:**

Refinement on *F*^2^	49 parameters
Least-squares matrix: full	0 restraints
*R*[*F*^2^ > 2σ(*F*^2^)] = 0.020	*w* = 1/[σ^2^(*F*_o_^2^) + (0.0157*P*)^2^ + 3.707*P*] where *P* = (*F*_o_^2^ + 2*F*_c_^2^)/3
*wR*(*F*^2^) = 0.047	(Δ/σ)_max_ = 0.001
*S* = 1.15	Δρ_max_ = 1.18 e Å^−^^3^
790 reflections	Δρ_min_ = −0.87 e Å^−^^3^

### Trisilver iodide sulfate (Ag3ISO4) Special details

**Table:**

Geometry. All esds (except the esd in the dihedral angle between two l.s. planes) are estimated using the full covariance matrix. The cell esds are taken into account individually in the estimation of esds in distances, angles and torsion angles; correlations between esds in cell parameters are only used when they are defined by crystal symmetry. An approximate (isotropic) treatment of cell esds is used for estimating esds involving l.s. planes.

### Trisilver iodide sulfate (Ag3ISO4) Fractional atomic coordinates and isotropic or equivalent isotropic displacement parameters (Å^2^)

**Table:**

	*x*	*y*	*z*	*U*_iso_*/*U*_eq_	
Ag1	0.32585 (4)	0.52882 (5)	0.31134 (4)	0.03257 (12)	
Ag2	0.36026 (6)	0.250000	0.61963 (5)	0.02763 (13)	
S1	0.13364 (15)	0.250000	0.10724 (13)	0.0164 (3)	
O1	0.1902 (3)	0.0758 (4)	0.0410 (3)	0.0241 (6)	
O2	0.4673 (4)	0.250000	0.3981 (4)	0.0228 (9)	
O3	0.1830 (5)	0.250000	0.2443 (4)	0.0261 (9)	
I1	0.00623 (4)	0.250000	0.63835 (4)	0.02701 (12)	

### Trisilver iodide sulfate (Ag3ISO4) Atomic displacement parameters (Å^2^)

**Table:**

	*U*^11^	*U*^22^	*U*^33^	*U*^12^	*U*^13^	*U*^23^
Ag1	0.0456 (2)	0.0224 (2)	0.0297 (2)	−0.00483 (15)	−0.00981 (15)	0.00358 (13)
Ag2	0.0364 (3)	0.0214 (2)	0.0251 (2)	0.000000	0.00360 (19)	0.000000
S1	0.0173 (6)	0.0124 (6)	0.0196 (6)	0.000000	−0.0023 (5)	0.000000
O1	0.0279 (15)	0.0149 (14)	0.0296 (16)	0.0039 (12)	0.0007 (12)	−0.0033 (12)
O2	0.0167 (19)	0.022 (2)	0.029 (2)	0.000000	0.0022 (17)	0.000000
O3	0.032 (2)	0.025 (2)	0.021 (2)	0.000000	−0.0065 (18)	0.000000
I1	0.0207 (2)	0.0343 (2)	0.0260 (2)	0.000000	−0.00100 (15)	0.000000

### Trisilver iodide sulfate (Ag3ISO4) Geometric parameters (Å, º)

**Table:**

Ag1—O1^i^	2.384 (3)	Ag2—O1^i^	2.436 (3)
Ag1—O3	2.413 (3)	Ag2—O2	2.467 (4)
Ag1—O2	2.473 (3)	Ag2—I1^vi^	2.8066 (7)
Ag1—I1^ii^	2.7838 (5)	Ag2—I1	3.1714 (7)
Ag1—Ag2^ii^	2.9973 (6)	S1—O1^vii^	1.474 (3)
Ag1—Ag1^iii^	3.0604 (8)	S1—O1	1.474 (3)
Ag1—Ag2^iv^	3.2744 (6)	S1—O3	1.475 (4)
Ag2—O1^v^	2.436 (3)	S1—O2^viii^	1.488 (4)
			
O1^i^—Ag1—O3	111.03 (13)	Ag1^x^—Ag2—I1	53.562 (13)
O1^i^—Ag1—O2	77.35 (12)	O1^v^—Ag2—Ag1^iv^	131.52 (7)
O3—Ag1—O2	75.51 (11)	O1^i^—Ag2—Ag1^iv^	78.36 (7)
O1^i^—Ag1—I1^ii^	126.05 (7)	O2—Ag2—Ag1^iv^	82.36 (9)
O3—Ag1—I1^ii^	122.87 (11)	I1^vi^—Ag2—Ag1^iv^	53.825 (13)
O2—Ag1—I1^ii^	112.50 (9)	Ag1^ix^—Ag2—Ag1^iv^	95.464 (13)
O1^i^—Ag1—Ag2^ii^	123.11 (7)	Ag1^x^—Ag2—Ag1^iv^	124.916 (17)
O3—Ag1—Ag2^ii^	85.82 (8)	I1—Ag2—Ag1^iv^	147.406 (12)
O2—Ag1—Ag2^ii^	156.75 (9)	O1^v^—Ag2—Ag1^xi^	78.36 (7)
I1^ii^—Ag1—Ag2^ii^	66.419 (15)	O1^i^—Ag2—Ag1^xi^	131.52 (7)
O1^i^—Ag1—Ag1^iii^	82.17 (7)	O2—Ag2—Ag1^xi^	82.36 (9)
O3—Ag1—Ag1^iii^	143.06 (8)	I1^vi^—Ag2—Ag1^xi^	53.825 (13)
O2—Ag1—Ag1^iii^	141.27 (8)	Ag1^ix^—Ag2—Ag1^xi^	124.916 (17)
I1^ii^—Ag1—Ag1^iii^	56.657 (9)	Ag1^x^—Ag2—Ag1^xi^	95.464 (13)
Ag2^ii^—Ag1—Ag1^iii^	59.302 (9)	I1—Ag2—Ag1^xi^	147.406 (12)
O1^i^—Ag1—Ag2^iv^	76.94 (7)	Ag1^iv^—Ag2—Ag1^xi^	55.720 (16)
O3—Ag1—Ag2^iv^	152.71 (9)	O1^vii^—S1—O1	109.7 (3)
O2—Ag1—Ag2^iv^	81.26 (8)	O1^vii^—S1—O3	109.73 (16)
I1^ii^—Ag1—Ag2^iv^	54.469 (13)	O1—S1—O3	109.73 (16)
Ag2^ii^—Ag1—Ag2^iv^	112.299 (13)	O1^vii^—S1—O2^viii^	109.04 (16)
Ag1^iii^—Ag1—Ag2^iv^	62.141 (8)	O1—S1—O2^viii^	109.04 (16)
O1^v^—Ag2—O1^i^	135.36 (14)	O3—S1—O2^viii^	109.5 (3)
O1^v^—Ag2—O2	76.49 (8)	S1—O1—Ag1^xii^	123.18 (18)
O1^i^—Ag2—O2	76.49 (8)	S1—O1—Ag2^xiii^	122.67 (17)
O1^v^—Ag2—I1^vi^	112.31 (7)	Ag1^xii^—O1—Ag2^xiii^	100.99 (11)
O1^i^—Ag2—I1^vi^	112.31 (7)	S1^xiv^—O2—Ag2	114.9 (2)
O2—Ag2—I1^vi^	129.46 (10)	S1^xiv^—O2—Ag1^vii^	119.86 (13)
O1^v^—Ag2—Ag1^ix^	125.95 (7)	Ag2—O2—Ag1^vii^	97.68 (11)
O1^i^—Ag2—Ag1^ix^	69.06 (7)	S1^xiv^—O2—Ag1	119.86 (13)
O2—Ag2—Ag1^ix^	145.14 (4)	Ag2—O2—Ag1	97.68 (11)
I1^vi^—Ag2—Ag1^ix^	71.152 (15)	Ag1^vii^—O2—Ag1	102.54 (15)
O1^v^—Ag2—Ag1^x^	69.06 (7)	S1—O3—Ag1^vii^	115.50 (15)
O1^i^—Ag2—Ag1^x^	125.95 (7)	S1—O3—Ag1	115.50 (15)
O2—Ag2—Ag1^x^	145.14 (4)	Ag1^vii^—O3—Ag1	106.12 (16)
I1^vi^—Ag2—Ag1^x^	71.152 (15)	Ag1^ix^—I1—Ag1^x^	66.688 (19)
Ag1^ix^—Ag2—Ag1^x^	61.397 (17)	Ag1^ix^—I1—Ag2^xv^	71.707 (15)
O1^v^—Ag2—I1	80.51 (7)	Ag1^x^—I1—Ag2^xv^	71.707 (15)
O1^i^—Ag2—I1	80.51 (7)	Ag1^ix^—I1—Ag2	60.018 (13)
O2—Ag2—I1	116.30 (10)	Ag1^x^—I1—Ag2	60.018 (13)
I1^vi^—Ag2—I1	114.243 (18)	Ag2^xv^—I1—Ag2	121.190 (18)
Ag1^ix^—Ag2—I1	53.563 (13)		

Symmetry codes: (i) −*x*+1/2, *y*+1/2, *z*+1/2; (ii) −*x*+1/2, −*y*+1, *z*−1/2; (iii) *x*, −*y*+3/2, *z*; (iv) −*x*+1, −*y*+1, −*z*+1; (v) −*x*+1/2, −*y*, *z*+1/2; (vi) *x*+1/2, *y*, −*z*+3/2; (vii) *x*, −*y*+1/2, *z*; (viii) *x*−1/2, *y*, −*z*+1/2; (ix) −*x*+1/2, −*y*+1, *z*+1/2; (x) −*x*+1/2, *y*−1/2, *z*+1/2; (xi) −*x*+1, *y*−1/2, −*z*+1; (xii) −*x*+1/2, *y*−1/2, *z*−1/2; (xiii) −*x*+1/2, −*y*, *z*−1/2; (xiv) *x*+1/2, *y*, −*z*+1/2; (xv) *x*−1/2, *y*, −*z*+3/2.

### Tetrasilver diiodide sulfate (Ag4I2SO4) Crystal data

**Table:**

Ag_4_I_2_SO_4_	*D*_x_ = 6.188 Mg m^−^^3^
*M**_r_* = 781.34	Mo *K*α radiation, λ = 0.71073 Å
Orthorhombic, *P**n**a*2_1_	Cell parameters from 6005 reflections
*a* = 9.2072 (3) Å	θ = 2.7–27.5°
*b* = 13.1007 (4) Å	µ = 16.77 mm^−^^1^
*c* = 6.9528 (2) Å	*T* = 301 K
*V* = 838.65 (4) Å^3^	Platelet, translucent light colourless-brown
*Z* = 4	0.10 × 0.03 × 0.01 mm
*F*(000) = 1368	

### Tetrasilver diiodide sulfate (Ag4I2SO4) Data collection

**Table:**

Bruker APEXII CCD diffractometer	1806 independent reflections
Radiation source: micro focus sealed tube	1768 reflections with *I* > 2σ(*I*)
Detector resolution: 7.3910 pixels mm^-1^	*R*_int_ = 0.035
φ and ω scans	θ_max_ = 27.5°, θ_min_ = 2.7°
Absorption correction: multi-scan (*SADABS*; Krause *et al.*, 2015)	*h* = −11→11
*T*_min_ = 0.53, *T*_max_ = 0.75	*k* = −14→17
6220 measured reflections	*l* = −8→9

### Tetrasilver diiodide sulfate (Ag4I2SO4) Refinement

**Table:**

Refinement on *F*^2^	*w* = 1/[σ^2^(*F*_o_^2^) + (0.017*P*)^2^ + 1.4488*P*] where *P* = (*F*_o_^2^ + 2*F*_c_^2^)/3
Least-squares matrix: full	(Δ/σ)_max_ < 0.001
*R*[*F*^2^ > 2σ(*F*^2^)] = 0.022	Δρ_max_ = 0.93 e Å^−^^3^
*wR*(*F*^2^) = 0.051	Δρ_min_ = −0.79 e Å^−^^3^
*S* = 1.08	Extinction correction: SHELXL-2017/1 (Sheldrick 2015b)
1806 reflections	Extinction coefficient: 0.00063 (15)
115 parameters	Absolute structure: Refined as an inversion twin
2 restraints	Absolute structure parameter: 0.35 (4)

### Tetrasilver diiodide sulfate (Ag4I2SO4) Special details

**Table:**

Geometry. All esds (except the esd in the dihedral angle between two l.s. planes) are estimated using the full covariance matrix. The cell esds are taken into account individually in the estimation of esds in distances, angles and torsion angles; correlations between esds in cell parameters are only used when they are defined by crystal symmetry. An approximate (isotropic) treatment of cell esds is used for estimating esds involving l.s. planes.
Refinement. Refined as a 2-component inversion twin.

### Tetrasilver diiodide sulfate (Ag4I2SO4) Fractional atomic coordinates and isotropic or equivalent isotropic displacement parameters (Å^2^)

**Table:**

	*x*	*y*	*z*	*U*_iso_*/*U*_eq_	Occ. (<1)
Ag1A	0.0380 (2)	0.24220 (17)	0.0000 (8)	0.0529 (8)	0.742 (10)
Ag1B	0.0376 (7)	0.2412 (6)	0.0855 (16)	0.0529 (8)	0.258 (10)
Ag2	0.11553 (8)	0.58050 (6)	0.06521 (13)	0.04291 (19)	
Ag3A	0.2608 (2)	0.31320 (14)	0.4098 (4)	0.0525 (4)	0.803 (4)
Ag3B	0.2122 (16)	0.3217 (11)	0.379 (3)	0.0525 (4)	0.098 (4)
Ag3C	0.2674 (19)	0.3129 (13)	0.480 (3)	0.0525 (4)	0.099 (4)
Ag4	0.48770 (11)	0.48881 (8)	0.31016 (16)	0.0547 (3)	
S1	0.31461 (18)	0.07193 (12)	0.0636 (3)	0.0208 (3)	
O1	0.1337 (7)	0.6252 (5)	0.3912 (9)	0.0317 (14)	
O2	0.1397 (6)	0.4646 (4)	0.5663 (12)	0.0350 (13)	
O3	0.1513 (5)	0.0756 (4)	0.0660 (10)	0.0263 (11)	
O4	0.3705 (7)	0.1259 (5)	0.2334 (10)	0.0337 (15)	
I1	0.04299 (6)	0.19055 (4)	0.57387 (12)	0.03413 (16)	
I2	0.26710 (6)	0.38893 (4)	0.03359 (10)	0.03490 (16)	

### Tetrasilver diiodide sulfate (Ag4I2SO4) Atomic displacement parameters (Å^2^)

**Table:**

	*U*^11^	*U*^22^	*U*^33^	*U*^12^	*U*^13^	*U*^23^
Ag1A	0.0359 (4)	0.0306 (4)	0.092 (2)	0.0056 (3)	−0.0114 (10)	0.0028 (11)
Ag1B	0.0359 (4)	0.0306 (4)	0.092 (2)	0.0056 (3)	−0.0114 (10)	0.0028 (11)
Ag2	0.0453 (4)	0.0525 (4)	0.0309 (4)	0.0006 (3)	−0.0006 (3)	−0.0021 (4)
Ag3A	0.0425 (8)	0.0608 (7)	0.0541 (11)	−0.0027 (6)	0.0023 (10)	0.0136 (8)
Ag3B	0.0425 (8)	0.0608 (7)	0.0541 (11)	−0.0027 (6)	0.0023 (10)	0.0136 (8)
Ag3C	0.0425 (8)	0.0608 (7)	0.0541 (11)	−0.0027 (6)	0.0023 (10)	0.0136 (8)
Ag4	0.0615 (5)	0.0574 (5)	0.0452 (5)	0.0062 (4)	0.0188 (4)	−0.0102 (4)
S1	0.0196 (8)	0.0210 (7)	0.0218 (8)	−0.0008 (6)	0.0004 (7)	−0.0001 (9)
O1	0.036 (3)	0.036 (4)	0.023 (3)	0.005 (3)	−0.006 (2)	0.001 (3)
O2	0.029 (3)	0.025 (2)	0.051 (4)	−0.004 (2)	0.004 (3)	0.003 (4)
O3	0.019 (2)	0.030 (2)	0.030 (3)	0.0000 (19)	0.004 (3)	0.008 (3)
O4	0.036 (3)	0.036 (4)	0.029 (3)	−0.001 (3)	−0.005 (3)	−0.005 (3)
I1	0.0326 (3)	0.0300 (3)	0.0397 (3)	−0.00035 (19)	0.0040 (3)	0.0033 (3)
I2	0.0268 (3)	0.0339 (3)	0.0440 (4)	0.00010 (18)	0.0011 (3)	0.0008 (3)

### Tetrasilver diiodide sulfate (Ag4I2SO4) Geometric parameters (Å, º)

**Table:**

Ag1A—Ag1B	0.595 (7)	Ag3A—I1^v^	2.838 (3)
Ag1A—O3	2.462 (5)	Ag3A—Ag4	3.184 (2)
Ag1A—O1^i^	2.468 (7)	Ag3B—Ag3C	0.88 (3)
Ag1A—I2	2.863 (2)	Ag3B—O2	2.375 (18)
Ag1A—I2^ii^	3.038 (2)	Ag3B—I2	2.608 (17)
Ag1A—I1^iii^	3.039 (5)	Ag3B—I1	2.686 (15)
Ag1A—Ag3B	3.256 (18)	Ag3C—O2	2.385 (19)
Ag1B—O3	2.412 (8)	Ag3C—I1^v^	2.620 (17)
Ag1B—O4^ii^	2.541 (10)	Ag3C—I1	2.694 (16)
Ag1B—Ag3B	2.805 (19)	Ag3C—I2	3.263 (17)
Ag1B—I2	2.889 (7)	Ag3C—Ag4	3.290 (16)
Ag1B—I2^ii^	3.039 (7)	Ag4—O3^v^	2.421 (6)
Ag1B—Ag3A	3.194 (10)	Ag4—O3^vi^	2.469 (7)
Ag2—O1	2.347 (6)	Ag4—I1^v^	3.0237 (13)
Ag2—O4^iv^	2.386 (7)	Ag4—I2	3.0879 (13)
Ag2—O2^i^	2.423 (5)	Ag4—I1^iv^	3.1247 (12)
Ag2—I2	2.8799 (9)	Ag4—I2^vii^	3.1741 (13)
Ag2—Ag3C^iv^	3.283 (18)	S1—O1^viii^	1.466 (6)
Ag3A—Ag3B	0.508 (14)	S1—O2^viii^	1.468 (5)
Ag3A—O2	2.522 (6)	S1—O4	1.469 (7)
Ag3A—I2	2.798 (3)	S1—O3	1.505 (5)
Ag3A—I1	2.8113 (19)		
			
Ag1B—Ag1A—O3	78.3 (9)	I2—Ag4—I1^iv^	88.37 (3)
Ag1B—Ag1A—O1^i^	108.5 (9)	O3^v^—Ag4—I2^vii^	94.40 (13)
O3—Ag1A—O1^i^	162.3 (2)	O3^vi^—Ag4—I2^vii^	77.50 (12)
Ag1B—Ag1A—I2	86.5 (9)	I1^v^—Ag4—I2^vii^	88.60 (3)
O3—Ag1A—I2	105.54 (14)	I2—Ag4—I2^vii^	170.55 (4)
O1^i^—Ag1A—I2	91.39 (17)	I1^iv^—Ag4—I2^vii^	83.97 (3)
Ag1B—Ag1A—I2^ii^	84.5 (9)	O3^v^—Ag4—Ag3A	107.99 (13)
O3—Ag1A—I2^ii^	80.34 (13)	O3^vi^—Ag4—Ag3A	80.56 (12)
O1^i^—Ag1A—I2^ii^	84.01 (16)	I1^v^—Ag4—Ag3A	54.34 (5)
I2—Ag1A—I2^ii^	168.03 (17)	I2—Ag4—Ag3A	52.97 (5)
Ag1B—Ag1A—I1^iii^	165.8 (9)	I1^iv^—Ag4—Ag3A	131.78 (6)
O3—Ag1A—I1^iii^	88.7 (2)	I2^vii^—Ag4—Ag3A	136.45 (6)
O1^i^—Ag1A—I1^iii^	82.4 (2)	O3^v^—Ag4—Ag3C	113.0 (3)
I2—Ag1A—I1^iii^	102.59 (13)	O3^vi^—Ag4—Ag3C	75.2 (3)
I2^ii^—Ag1A—I1^iii^	87.79 (10)	I1^v^—Ag4—Ag3C	48.8 (3)
Ag1B—Ag1A—Ag3B	37.0 (9)	I2—Ag4—Ag3C	61.4 (3)
O3—Ag1A—Ag3B	85.6 (3)	I1^iv^—Ag4—Ag3C	136.5 (3)
O1^i^—Ag1A—Ag3B	109.8 (3)	I2^vii^—Ag4—Ag3C	128.0 (3)
I2—Ag1A—Ag3B	49.9 (3)	Ag3A—Ag4—Ag3C	8.6 (3)
I2^ii^—Ag1A—Ag3B	121.5 (3)	O1^viii^—S1—O2^viii^	111.9 (4)
I1^iii^—Ag1A—Ag3B	148.5 (3)	O1^viii^—S1—O4	108.3 (3)
Ag1A—Ag1B—O3	87.8 (9)	O2^viii^—S1—O4	110.5 (4)
Ag1A—Ag1B—O4^ii^	113.2 (9)	O1^viii^—S1—O3	108.6 (4)
O3—Ag1B—O4^ii^	154.4 (5)	O2^viii^—S1—O3	108.5 (3)
Ag1A—Ag1B—Ag3B	135.6 (10)	O4—S1—O3	109.0 (4)
O3—Ag1B—Ag3B	97.5 (4)	S1^vi^—O1—Ag2	134.0 (4)
O4^ii^—Ag1B—Ag3B	78.2 (4)	S1^vi^—O1—Ag1A^x^	107.0 (4)
Ag1A—Ag1B—I2	81.6 (9)	Ag2—O1—Ag1A^x^	115.2 (3)
O3—Ag1B—I2	106.1 (3)	S1^vi^—O2—Ag3B	131.9 (5)
O4^ii^—Ag1B—I2	92.0 (3)	S1^vi^—O2—Ag3C	130.8 (5)
Ag3B—Ag1B—I2	54.5 (3)	Ag3B—O2—Ag3C	21.2 (6)
Ag1A—Ag1B—I2^ii^	84.3 (9)	S1^vi^—O2—Ag2^x^	120.8 (3)
O3—Ag1B—I2^ii^	81.1 (2)	Ag3B—O2—Ag2^x^	94.5 (4)
O4^ii^—Ag1B—I2^ii^	86.4 (2)	Ag3C—O2—Ag2^x^	105.9 (5)
Ag3B—Ag1B—I2^ii^	140.1 (5)	S1^vi^—O2—Ag3A	128.4 (4)
I2—Ag1B—I2^ii^	163.8 (4)	Ag3B—O2—Ag3A	11.4 (4)
Ag1A—Ag1B—Ag3A	134.0 (10)	Ag3C—O2—Ag3A	11.1 (4)
O3—Ag1B—Ag3A	91.5 (3)	Ag2^x^—O2—Ag3A	103.6 (2)
O4^ii^—Ag1B—Ag3A	84.3 (3)	S1—O3—Ag1B	117.6 (3)
Ag3B—Ag1B—Ag3A	6.3 (3)	S1—O3—Ag4^ii^	128.2 (4)
I2—Ag1B—Ag3A	54.51 (14)	Ag1B—O3—Ag4^ii^	90.2 (3)
I2^ii^—Ag1B—Ag3A	141.0 (4)	S1—O3—Ag1A	116.7 (3)
O1—Ag2—O4^iv^	150.21 (19)	Ag1B—O3—Ag1A	13.97 (18)
O1—Ag2—O2^i^	97.3 (3)	Ag4^ii^—O3—Ag1A	100.2 (2)
O4^iv^—Ag2—O2^i^	96.7 (3)	S1—O3—Ag4^viii^	119.7 (4)
O1—Ag2—I2	104.87 (16)	Ag1B—O3—Ag4^viii^	103.3 (3)
O4^iv^—Ag2—I2	96.73 (16)	Ag4^ii^—O3—Ag4^viii^	91.27 (16)
O2^i^—Ag2—I2	104.94 (13)	Ag1A—O3—Ag4^viii^	93.1 (2)
O1—Ag2—Ag3C^iv^	85.3 (4)	S1—O4—Ag2^ix^	132.5 (4)
O4^iv^—Ag2—Ag3C^iv^	65.0 (3)	S1—O4—Ag1B^v^	102.5 (4)
O2^i^—Ag2—Ag3C^iv^	123.0 (3)	Ag2^ix^—O4—Ag1B^v^	122.0 (4)
I2—Ag2—Ag3C^iv^	129.4 (3)	Ag3C^ii^—I1—Ag3B	116.6 (6)
Ag3B—Ag3A—O2	68 (2)	Ag3C^ii^—I1—Ag3C	133.9 (5)
Ag3B—Ag3A—I2	63 (2)	Ag3B—I1—Ag3C	18.7 (5)
O2—Ag3A—I2	97.68 (19)	Ag3C^ii^—I1—Ag3A	126.9 (4)
Ag3B—Ag3A—I1	70.6 (18)	Ag3B—I1—Ag3A	10.3 (3)
O2—Ag3A—I1	87.66 (14)	Ag3C—I1—Ag3A	10.0 (4)
I2—Ag3A—I1	126.63 (9)	Ag3C^ii^—I1—Ag3A^ii^	9.3 (4)
Ag3B—Ag3A—I1^v^	168.1 (17)	Ag3B—I1—Ag3A^ii^	109.8 (3)
O2—Ag3A—I1^v^	104.18 (17)	Ag3C—I1—Ag3A^ii^	128.0 (4)
I2—Ag3A—I1^v^	111.27 (7)	Ag3A—I1—Ag3A^ii^	119.99 (8)
I1—Ag3A—I1^v^	118.69 (9)	Ag3C^ii^—I1—Ag4^ii^	70.9 (4)
Ag3B—Ag3A—Ag4	109.1 (17)	Ag3B—I1—Ag4^ii^	106.8 (4)
O2—Ag3A—Ag4	79.38 (13)	Ag3C—I1—Ag4^ii^	116.4 (4)
I2—Ag3A—Ag4	61.75 (5)	Ag3A—I1—Ag4^ii^	108.56 (5)
I1—Ag3A—Ag4	165.76 (9)	Ag3A^ii^—I1—Ag4^ii^	65.71 (4)
I1^v^—Ag3A—Ag4	59.95 (5)	Ag3C^ii^—I1—Ag1A^xi^	103.4 (4)
Ag3B—Ag3A—Ag1B	37 (2)	Ag3B—I1—Ag1A^xi^	111.0 (4)
O2—Ag3A—Ag1B	104.6 (2)	Ag3C—I1—Ag1A^xi^	96.5 (4)
I2—Ag3A—Ag1B	57.19 (18)	Ag3A—I1—Ag1A^xi^	106.20 (7)
I1—Ag3A—Ag1B	70.03 (18)	Ag3A^ii^—I1—Ag1A^xi^	112.45 (6)
I1^v^—Ag3A—Ag1B	150.21 (16)	Ag4^ii^—I1—Ag1A^xi^	139.61 (5)
Ag4—Ag3A—Ag1B	118.82 (19)	Ag3C^ii^—I1—Ag4^ix^	91.6 (4)
Ag3A—Ag3B—Ag3C	29 (2)	Ag3B—I1—Ag4^ix^	149.2 (3)
Ag3A—Ag3B—O2	101 (2)	Ag3C—I1—Ag4^ix^	134.4 (4)
Ag3C—Ag3B—O2	80.0 (16)	Ag3A—I1—Ag4^ix^	139.57 (5)
Ag3A—Ag3B—I2	107 (2)	Ag3A^ii^—I1—Ag4^ix^	96.54 (5)
Ag3C—Ag3B—I2	132.3 (15)	Ag4^ii^—I1—Ag4^ix^	69.285 (12)
O2—Ag3B—I2	107.1 (6)	Ag1A^xi^—I1—Ag4^ix^	71.00 (5)
Ag3A—Ag3B—I1	99.1 (19)	Ag3B—I2—Ag3A	10.0 (3)
Ag3C—Ag3B—I1	81.2 (14)	Ag3B—I2—Ag1A	72.9 (4)
O2—Ag3B—I1	93.7 (6)	Ag3A—I2—Ag1A	79.80 (12)
I2—Ag3B—I1	142.4 (7)	Ag3B—I2—Ag2	97.5 (3)
Ag3A—Ag3B—Ag1B	137 (2)	Ag3A—I2—Ag2	103.15 (5)
Ag3C—Ag3B—Ag1B	150.2 (19)	Ag1A—I2—Ag2	103.55 (5)
O2—Ag3B—Ag1B	122.3 (6)	Ag3B—I2—Ag1B	61.1 (4)
I2—Ag3B—Ag1B	64.4 (4)	Ag3A—I2—Ag1B	68.3 (2)
I1—Ag3B—Ag1B	78.1 (4)	Ag1A—I2—Ag1B	11.86 (15)
Ag3A—Ag3B—Ag1A	135 (2)	Ag2—I2—Ag1B	102.70 (14)
Ag3C—Ag3B—Ag1A	153.4 (19)	Ag3B—I2—Ag1A^v^	92.2 (3)
O2—Ag3B—Ag1A	123.9 (6)	Ag3A—I2—Ag1A^v^	83.59 (11)
I2—Ag3B—Ag1A	57.2 (4)	Ag1A—I2—Ag1A^v^	102.64 (3)
I1—Ag3B—Ag1A	85.3 (4)	Ag2—I2—Ag1A^v^	153.71 (5)
Ag1B—Ag3B—Ag1A	7.34 (19)	Ag1B—I2—Ag1A^v^	103.38 (17)
Ag3B—Ag3C—O2	78.8 (15)	Ag3B—I2—Ag1B^v^	82.0 (4)
Ag3B—Ag3C—I1^v^	139.8 (16)	Ag3A—I2—Ag1B^v^	73.0 (2)
O2—Ag3C—I1^v^	115.5 (7)	Ag1A—I2—Ag1B^v^	103.69 (16)
Ag3B—Ag3C—I1	80.1 (14)	Ag2—I2—Ag1B^v^	151.25 (14)
O2—Ag3C—I1	93.3 (6)	Ag1B—I2—Ag1B^v^	102.05 (7)
I1^v^—Ag3C—I1	132.3 (7)	Ag1A^v^—I2—Ag1B^v^	11.23 (14)
Ag3B—Ag3C—I2	36.3 (13)	Ag3B—I2—Ag4	72.4 (3)
O2—Ag3C—I2	89.1 (5)	Ag3A—I2—Ag4	65.28 (5)
I1^v^—Ag3C—I2	104.0 (5)	Ag1A—I2—Ag4	145.07 (11)
I1—Ag3C—I2	114.2 (6)	Ag2—I2—Ag4	84.37 (3)
Ag3B—Ag3C—Ag2^ix^	117.2 (18)	Ag1B—I2—Ag4	133.4 (2)
O2—Ag3C—Ag2^ix^	152.8 (7)	Ag1A^v^—I2—Ag4	75.37 (8)
I1^v^—Ag3C—Ag2^ix^	67.8 (4)	Ag1B^v^—I2—Ag4	67.96 (17)
I1—Ag3C—Ag2^ix^	69.9 (4)	Ag3B—I2—Ag4^xii^	139.4 (3)
I2—Ag3C—Ag2^ix^	117.0 (5)	Ag3A—I2—Ag4^xii^	130.63 (5)
Ag3B—Ag3C—Ag4	88.6 (15)	Ag1A—I2—Ag4^xii^	145.36 (11)
O2—Ag3C—Ag4	79.1 (5)	Ag2—I2—Ag4^xii^	86.69 (3)
I1^v^—Ag3C—Ag4	60.3 (3)	Ag1B—I2—Ag4^xii^	156.9 (2)
I1—Ag3C—Ag4	167.4 (7)	Ag1A^v^—I2—Ag4^xii^	70.35 (7)
I2—Ag3C—Ag4	56.2 (3)	Ag1B^v^—I2—Ag4^xii^	76.02 (16)
Ag2^ix^—Ag3C—Ag4	120.8 (5)	Ag4—I2—Ag4^xii^	67.860 (11)
O3^v^—Ag4—O3^vi^	171.17 (7)	Ag3B—I2—Ag3C	11.5 (5)
O3^v^—Ag4—I1^v^	92.83 (15)	Ag3A—I2—Ag3C	3.2 (3)
O3^vi^—Ag4—I1^v^	90.48 (13)	Ag1A—I2—Ag3C	82.7 (3)
O3^v^—Ag4—I2	79.91 (14)	Ag2—I2—Ag3C	101.2 (3)
O3^vi^—Ag4—I2	107.64 (13)	Ag1B—I2—Ag3C	71.2 (4)
I1^v^—Ag4—I2	99.15 (3)	Ag1A^v^—I2—Ag3C	84.3 (3)
O3^v^—Ag4—I1^iv^	89.01 (14)	Ag1B^v^—I2—Ag3C	73.4 (4)
O3^vi^—Ag4—I1^iv^	86.69 (13)	Ag4—I2—Ag3C	62.3 (3)
I1^v^—Ag4—I1^iv^	172.46 (4)	Ag4^xii^—I2—Ag3C	128.3 (3)

Symmetry codes: (i) −*x*, −*y*+1, *z*−1/2; (ii) *x*−1/2, −*y*+1/2, *z*; (iii) *x*, *y*, *z*−1; (iv) −*x*+1/2, *y*+1/2, *z*−1/2; (v) *x*+1/2, −*y*+1/2, *z*; (vi) −*x*+1/2, *y*+1/2, *z*+1/2; (vii) −*x*+1, −*y*+1, *z*+1/2; (viii) −*x*+1/2, *y*−1/2, *z*−1/2; (ix) −*x*+1/2, *y*−1/2, *z*+1/2; (x) −*x*, −*y*+1, *z*+1/2; (xi) *x*, *y*, *z*+1; (xii) −*x*+1, −*y*+1, *z*−1/2.
